# Impulsivity during adolescent development: Insights from a decade of ABCD study longitudinal analyses

**DOI:** 10.1016/j.dcn.2026.101832

**Published:** 2026-10-06

**Authors:** Nioud Mulugeta Gebru, Priscila Dib Gonçalves, Faith Adams, Nicita Anand, Laura N. Haygood, Micah E. Johnson, Alexandra O. Cohen

**Affiliations:** a Department of Family and Preventive Medicine, Emory University, Atlanta, GA, USA; b Department of Psychiatry, Columbia University Irving Medical Center, New York, NY, USA; c Department of Psychiatry, Icahn School of Medicine at Mount Sinai, New York, NY, USA; d Brown University Library, Providence, RI, USA; e Department of Family Medicine, Geffen School of Medicine, University of California, Los Angeles, CA, USA; f Department of Psychology, Emory University, Atlanta, GA, USA

**Keywords:** ABCD study, Impulsivity, Adolescence, Trait impulsivity, Behavior, Neurodevelopment

## Abstract

**Background:**

The Adolescent Brain Cognitive Development (ABCD) study provides an unprecedented opportunity to examine how impulsivity develops and predicts health outcomes. This review synthesized longitudinal findings from the study’s first decade and identified priories for future research.

**Methods:**

Following Cochrane’s guidelines, we systematically searched for peer-reviewed papers published through July 2025. Eligible studies included self-reported or task-based measures of impulsivity and used two or more data waves. Fifty-one studies met inclusion criteria.

**Results:**

Impulsivity was predominantly operationalized using self-report trait measures, particularly the UPPS-P or urgency-specific measures (k = 42) and BIS/BAS scales (k = 23); task-based measures used less often (k = 20). Across health domains, urgency, sensation seeking, and lack of premeditation (impulse control) showed recurring prospective associations, although findings varied across outcomes and studies. Findings involving task-based measures also varied across studies. Several studies examined impulsivity as an outcome of environmental exposures, supporting its developmental malleability, but few explicitly modeled changes in impulsivity over time. Few eligible studies examined neuroimaging measures, precluding firm conclusions about the magnitude or consistency of their associations.

**Conclusions:**

Findings from ABCD extend prior impulsivity research by demonstrating that multiple self-reported impulsivity facets are early-emerging prospective correlates of health risk in a large preadolescent cohort. Divergence between self-report and task-based findings may reflect the multi-dimensionality of impulsivity and related constructs and highlights the need for standardized scoring across ABCD studies. The most important gap is the underutilization of ABCD’s longitudinal design to model trajectories of impulsivity itself. As additional waves become available, the field is well-positioned to move from establishing associations to characterizing developmental trajectories, mechanisms, and modifiable influences.

## Highlights

• ABCD data replicate prior findings of impulsivity as an early transdiagnostic risk factor.
• Task-based impulsivity shows weaker, less consistent effects than self-report measures.
• Most studies treat impulsivity as stable; trajectory modeling remains a critical gap.
• Replicable but small effects warrant caution against deterministic risk interpretation.

## 1 Introduction

The Adolescent Brain Cognitive Development (ABCD) study — which follows 11,878 children from ages 9–10 years into adolescence — is the largest longitudinal study of brain and behavioral development in the United States (Karcher and Barch, 2021) and presents an unprecedented opportunity to examine how impulsivity develops, what shapes it, and how it predicts health outcomes across a large cohort. ABCD conducts annual assessments with youth and their parents/guardians measuring a range of physical and mental health outcomes, including eating disorders, suicide risk, and substance use intentions and behaviors; as well as multiple well-validated measures of impulsivity that are self-report and task-based. A decade into the ABCD study, impulsivity-related findings remain dispersed across subdisciplines and measurement approaches are highly heterogeneous. Impulsivity-related findings from ABCD study have also not yet been systematically reviewed to clarify patterns in this accumulating literature, which would reveal gaps and directions for future analyses, further shedding light on the development and consequences of impulsive behaviors.

Impulsivity is a multifaceted construct that is broadly characterized by tendencies toward action without adequate forethought, difficulty regulating responses to emotional stimuli, and preference for immediate over delayed rewards (Brewer and Potenza, 2008, Leeman et al., 2019, Moeller et al., 2001). The three conceptually distinct but empirically overlapping impulsivity domains often assessed in the literature are impulsive trait, impulsive action, and impulsive choice (MacKillop et al., 2016). The ABCD study includes validated measures spanning all three domains —self-report trait measures (i.e., Urgency, Premeditation, Perseverance, Sensation Seeking (UPPS-P) and the Behavioral Inhibition/Activation System (BIS/BAS) scales) along with task-based measures of impulsive action (i.e., Stop-Signal task, Flanker) and impulsive choice paradigms (i.e., delay discounting, cash choice) — making it uniquely positioned to examine convergent and divergent findings across measurement modalities within a single large developmental cohort.

Trait impulsivity is thought to reflect relatively stable individual differences in impulsive tendencies, capturing broad patterns of individuals’ behaviors across context and time. As such, it is referred to as impulsive personality traits and is assessed through self-report questionnaires. Two widely used assessments of trait impulsivity are the UPPS-P [Urgency, Premeditation, Perseverance, Sensation Seeking] Scale and the Behavioral Inhibition/Activation System (BIS/BAS) scales (Carver and White, 1994, Whiteside and Lynam, 2001). Together, these assessments have been widely adopted and validated among adolescent populations and consistently linked to developmental and clinical outcomes including substance use, emotional dysregulation, suicidality and eating disorders (Harrison et al., 2010, Hildebrandt et al., 2021, Mallorquí-Bagué et al., 2018). Emerging evidence suggests that impulsive traits as measured by BIS/BAS and UPPS-P facets such as sensation seeking and lack of premeditation are linked to functional connectivity, particularly across cerebro-cerebellar networks and frontostriatal reward circuits (Abdallah et al., 2020, Angelides et al., 2017). Neuroimaging studies also show functional engagement of the pre-supplementary motor area and inferior frontal gyrus are linked to response inhibition and impulsivity among adolescents (Kaiser et al., 2022, Lohse et al., 2023, Schaum et al., 2021).

Impulsive action reflects difficulties inhibiting initiated responses, which is assessed using behavioral tasks such as stop signal task (SST), go/no-go paradigms, wherein participants are asked to respond to specific signals (i.e., stimuli) by initiating (e.g., go) or withholding (e.g., no-go) certain actions (e.g., press keyboard) (Weafer et al., 2013). Indices derived from such measures (e.g., reaction time, correct number of go/no-go responses) are thought to indicate aspects (e.g., speed) of inhibitory processes and have been closely related to inhibitory control, attentional difficulties, and executive function (Brevers et al., 2012). Impulsive action measures are commonly used to study neural correlates of impulsivity, with research showing differential engagement of cortical-subcortical systems, including frontostriatal (e.g., anterior dorsomedial prefrontal cortex, frontal–basal ganglia networks) circuits linked to heightened impulsive action (Wang et al., 2016).

Impulsive choice is the third dimension, typically operationalized as a general behavioral tendency toward more immediate rewards (MacKillop et al., 2016). Choice impulsivity is most often captured using delay discounting tasks, wherein participants are asked to make a series of choices between receiving smaller vs larger amounts of money at varying delays. These intertemporal choices can be used to calculate a discounting rate, thought to indicate the extent to which future rewards are devalued, characterizing individuals’ tendency for more impulsive choices (Reynolds and Schiffbauer, 2004). Impulsive choice, specifically as assessed via discounting tasks with monetary rewards, is differentially associated with the activation of reward processing neural systems. For immediate responses (i.e., preference for smaller rewards), greater engagement of medial cortical areas such as the ventromedial prefrontal cortex and posterior cingulate cortex are observed; meanwhile, for delayed responses (i.e., preference for larger rewards) there is recruitment of lateral prefrontal regions, implicated in cognitive control (Scheres et al., 2013). Prior studies have consistently linked delay discounting with a range of maladaptive behaviors in adolescents and teenagers, including substance use, academic performance, and sex-related risky behaviors (Felton et al., 2020, Kahn et al., 2015).

Although the tripartite framework of trait, action, and choice provides an organizing structure for this review, it is important to acknowledge that not all constructs operationalized as impulsivity-related in the ABCD literature map cleanly onto these domains. A growing body of research has questioned whether “impulsivity” should be treated as a single unitary psychological construct, noting that impulsivity and related constructs (e.g., inhibitory control, reward sensitivity) have different predictive and clinical profiles and should not be used interchangeably (Strickland and Johnson, 2021, Joyner et al., 2021). Within the ABCD literature, such impulsivity-adjacent constructs are nonetheless routinely operationalized as impulsivity indicators, and documenting this heterogeneity was one goal of the current review. Despite this heterogeneity, the breadth of constructs examined under the impulsivity umbrella reflects a shared interest in advancing our understanding on how self-regulation of behavioral and emotional responding develops during adolescence and how failures in that self-regulation confer risk.

## 2 The current systematic review

As the number of publications using ABCD study data to examine impulsivity related constructs continues to grow, and more prospective data become available, there is a critical need (and opportunity) to examine emerging findings to determine the current status of the ABCD impulsivity literature as well as identify research gaps and future directions. Indeed, given the wide array of conceptualizations, measurements, and outcomes of focus when examining impulsivity, findings largely remain dispersed across subdisciplines. To date, no systematic review has synthesized ABCD-related findings to clarify how different dimensions of impulsivity have been operationalized.

One essential contribution of the ABCD study to the field of adolescent development, beyond the impulsivity literature, is to advance our evidence on cognitive development. Extant findings are largely based on cross-sectional analyses; the ABCD study encourages the use of repeated assessments of variables of interest, including impulsivity, in a large cohort dataset. Thus, the present systematic review identifies trends in methods, conceptualization, and findings across impulsivity domains and health outcomes. Findings are discussed in light of what the ABCD literature adds to, and where it challenges, prior work on adolescent impulsivity. At the time of the current systematic review, the ABCD data release 6.0 was available, including data from baseline through the 6-year follow-up, and impulsivity assessments were collected during the baseline wave and in even year follow ups (e.g., years 2 and 4).

## 3 Methods

The protocol for this review was pre-registered in the International Prospective Register for Systematic Reviews (PROSPERO) under the registration number CRD420251111978. A comprehensive search was developed by a research librarian using Ovid MEDLINE in July 2025. Given the focus on studies using ABCD study data, the search was anchored to the ABCD Study Research Publication List (abcdstudy.org/research-publications), as it existed at the time of the search, which comprised 1501 total papers. Of those, 1445 citations had PubMed Identifiers (PMIDs), which were incorporated directly into the search. The remaining 56 citations were identified manually and added to the screening list.

Within this ABCD Publication List corpus, papers were filtered using both construct-based keywords and measure or task-specific terms representing the concept of impulsivity. Construct-based terms included: impuls*, urgency, sensation seeking, perseverance, planning, premeditation, inhibition, disinhibition. Measure/Task-specific terms included UPPS, the Stop Signal Task, Flanker Task, Delay Discounting Task.

This search was peer reviewed by a second research librarian using the Peer Review of Electronic Search Strategies (PRESS) checklist (McGowan et al., 2016). During peer review, we determined that some potentially relevant studies described specific behavioral tasks without using general impulsivity terminology. We therefore expanded the strategy to include task-specific terms and impulsivity-adjacent terminology (e.g., risk taking task, reward responsiveness). The final search was conducted on May 20, 2026 incorporating both the original impulsivity terms and the added task terms and was rerun across the full ABCD Publication List corpus available through July 2025. All records retrieved by the final strategy were screened according to the prespecified eligibility criteria. The final strategy, which retained all terms from the initial search and the expanded terminology, resulted in additional 190 records, from which 4 additional papers were found to be eligible, bringing the total of eligible studies to 51 included studies. This corpus-anchored approach ensured that no ABCD-based publication was excluded due to keyword gaps. The supplemental procedure did not constitute a comprehensive update for literature published after the original search cutoff. The full search details, including terms and keywords, are available in supplemental materials.

### 3.1 Criteria for inclusion and strategies for this review

To be included in this review, studies needed to be published in a peer-reviewed journal, written in English, and use data from the ABCD study. All studies needed to (1) have included at least one measure of impulsivity, either self-report (i.e., UPPS-P, BIS/BAS) or task (i.e., SSRT, cash choice, delay discounting task, or flanker); (2) clearly analyzed impulsivity separately from other constructs (e.g., not subsumed within a broader ADHD or psychopathology composite), and (3) include data from two or more ABCD waves (i.e., cross-sectional studies were not eligible).

### 3.2 Study selection, data extraction, and risk of bias assessment

Two randomly assigned author pairs independently screened titles and abstracts and completed the full text review. Disagreements were resolved through discussion. Data extraction was completed in duplicate, also by a random combination of two authors, using Covidence software. Data extracted included information about participant characteristics, study methodology, impulsivity measure(s) used, analytic approach, and reported findings.

Risk of bias was assessed using the National Heart, Lung, and Blood Institute (NHLBI) Quality Assessment Tool, which has a tailored assessment tool for Observational Cohort and Cross-sectional studies, given the cohort design of the ABCD study. The cohort assessment tool has 14-items to rate bias across a range of criteria (e.g., research question clarity, sample description, temporal timeframe adequacy, exposure gradient [continues vs categorical]), which were adapted for the current review (e.g., did not assess bias based blinding criteria, given the secondary data analyses of the ABCD study). The bias assessment is provided in supplementary materials. Each study was rated as good, fair, or poor across each criterion by two combinations of authors, and disagreements were resolved through discussion.

### 3.3 Data coding and categorization

To organize findings, extracted studies were coded along several dimensions. Impulsivity measures were categorized according to the trait-action-choice framework. Self-reports (i.e., UPPS-P, BIS/BAS) were classified as trait-based; Stop-Signal, Go/No-Go, and Flanker tasks as action-based; and delay discounting and cash choice paradigms as choice-based. Studies were coded by the role of impulsivity in the analytic model (predictor, outcome, mediator, covariate/correlate) and by health domain (substance use, mental health/social risk environment, cognition/brain, physical health, sleep/screen use, and suicide risk).

## 4 Results

### 4.1 Results of the search

The search retrieved a total of 1501 studies that used ABCD study data, of which 246 unique articles included references to impulsivity. Following title and abstract screening, 160 full-text articles were reviewed to determine inclusion. Fifty-one studies met all criteria and are included in this review. Fig. 1 displays the PRISMA diagram.

**Fig. 1:**
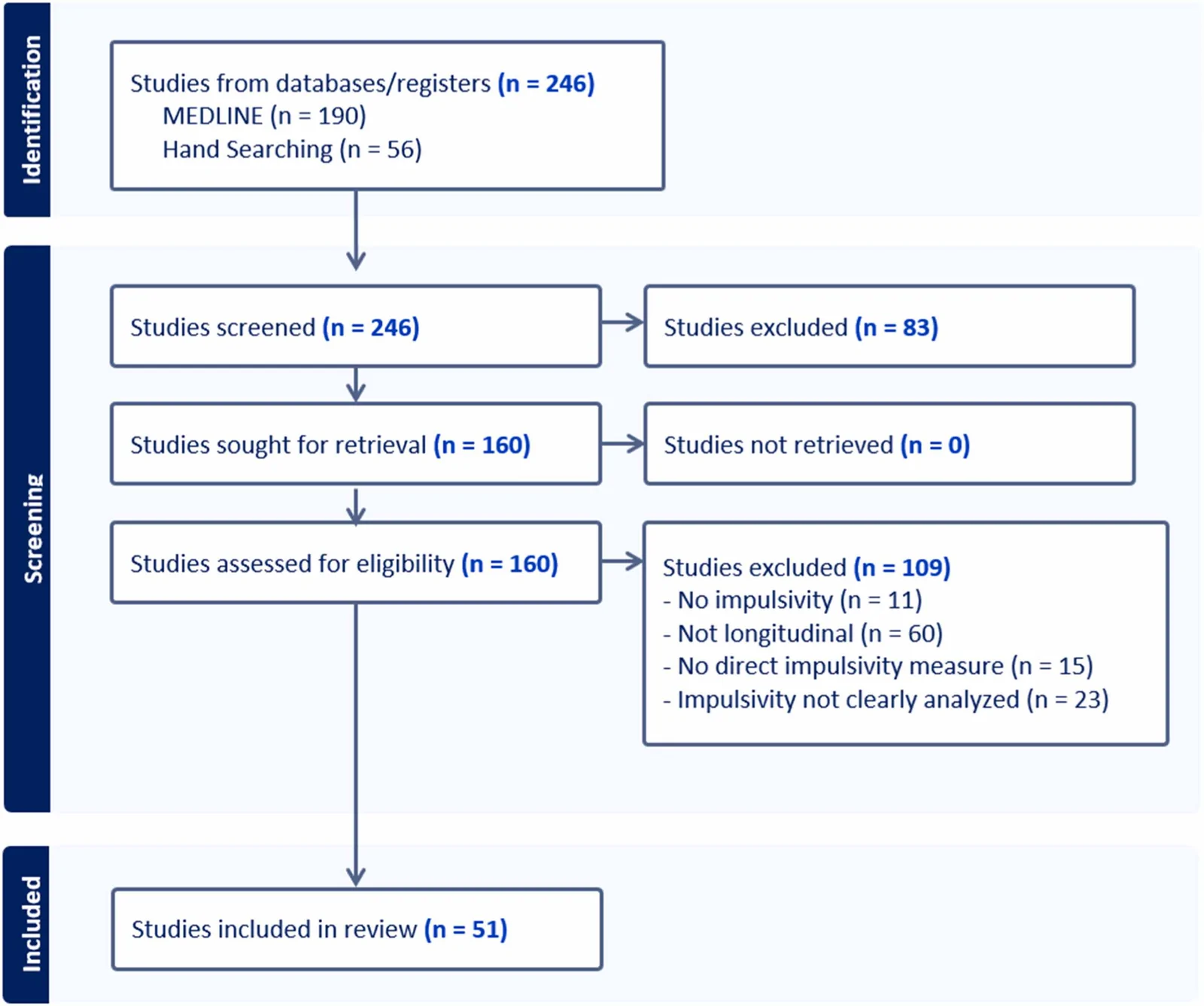
PRISMA flow diagram.

Studies were published between 2020 and 2025 and primarily leveraged baseline assessments and early follow-ups of the ABCD study data. Bias assessments rated 21 studies as good quality, 26 as fair, and 4 as poor based on NHLBI criteria (Table 1). Common strengths included large sample size, clear research aims, and well operationalized exposures/outcomes; common limitations included insufficient confounder adjustments and limited reporting of temporal relationships.

**Table 1:** Summary of included publications and reported findings on impulsivity.

**Study ID**	**Impulsivity Role**	**Type**	**Measures Used**	**Other Domain(s)**	**Direction of Findings**	**Risk of Bias**
Adams 2024	Predictor, Correlate	Trait, Choice, Action	UPPS-P, SST, CCT	Alcohol expectancies	↑ trait impulsivity, ↑ positive and ↓ negative alcohol expectancies	Good
Adise 2023a	Predictor, Outcome	Trait	UPPS-P, BIS/BAS	Weight gain, Brain structure	↑ UPPS Impulsivity → ↓ cortical thickness; ↑ weight gain → ↑ impulsivity	Fair
Adise 2023b	Predictor	Trait & Action	UPPS-P, BIS/BAS, Little Man, Flanker	Weight Gain, Cognition	↑ Impulsivity + ↓ inhibitory control → ↑ rate of BMI change from age 9–12	Fair
Adise 2024	Predictor	Trait & Action	UPPS-P, BIS/BAS, Little Man, Flanker	Diet intake (saturated fat/sugar)	↑ impulsivity + baseline BMI → ↑ increased intake at year 2	Fair
Agarwal 2025	Outcome	Trait	BIS/BAS, UPPS-P (urgency facets only)	Brain function, reward response, maladaptive behaviors	↑ childhood adversity/high risk status + ↓ cortical thickness/activation → ↑ impulsivity + behavioral problems	Fair
Amir 2025	Predictor, Mediator	Trait	BIS/BAS, UPPS-P	Psychotic-like experiences, neural response	Prenatal cannabis exposure → ↑ impulsivity/↓ neural response → ↑ Psychotic-like experiences	Poor
Chen 2025	Predictor	Trait	BIS/BAS, UPPS-P	Subthreshold depression	↑ Behavioral inhibition → ↑ risk MDD; ↑ lack of perseverance → ↑ persistence in subthreshold depression	Good
Christopher-Hayes 2024	Outcome	Action	Flanker Task	Inhibition/attention	Asthma history → ↓ episodic memory + ↓ processing speed + ↓ inhibition/attention	Fair
Correa 2023	Predictor	Trait	BIS/BAS	Anxiety & substance use	↑ BIS → ↑ anxiety/substance use	Good
Fan 2023	Correlate, Predictor	Trait	UPPS-P	Internalizing disorder subtypes & anxiety	Anxiety × Impulsivity Subtypes: Subtype 1: ↑ anxiety → ↑ lack of perseverance; Subtype 2: ↑ anxiety → ↓ sensation seeking; subtype 1 also showed ↑ cortical thickness, deficits, and emotional dysregulation	Fair
Ferariu 2024	Outcome	Trait, Action	UPPS-P, BIS/BAS, SST	Mental health & personality	↑ alcohol sipping → ↑ impulsivity & psychopathology trajectories	Fair
Gebru 2023	Outcome	Trait	UPPS-P	Parental mental health & Family conflict	↑ parental mental health → ↑ family conflict → ↑ impulsivity	Good
Gelino 2025	Predictor	Choice	Delay discounting	Sleep, obesity, psychopathology	↑ baseline discounting → ↑ sleep disturbance, waist circumference, externalizing psychopathology	Good
Gilman 2024a	Correlate	Trait	UPPS-P	Cannabis risk perception	↑ impulsivity → ↓ perceived cannabis harm	Fair
Gilman 2024b	Predictor, Correlate	Trait, Choice	UPPS-P, Delay discounting	Brain structure, polygenic risk scores	↑ impulsivity → ↓ cortical area (frontal/temporal); ↑ genetic risk → ↑ UPPS-P and ↓ cortical area/volume	Fair
Grund 2025	Predictor	Trait	UPPS-P, BIS/BAS	Screen use	↑ impulsivity → ↑ screen time	Good
Keyin 2025	Predictor	Trait	UPPS-P, BIS	Subthreshold anxiety	↑ impulsivity → ↑ anxiety risk	Fair
Kwon 2024	Predictor	Trait, Action	UPPS-P, BIS/BAS, SST	Soda intake, alcohol sipping	↑ impulsivity → ↑ soda intake and alcohol sipping	Fair
Lahey 2022	Mediator	Trait, Action	UPPS-P, Flanker Task	Psychological problems	↑ ADHD PRS → ↑ impulsivity → ↑ psychological problems	Fair
Lees 2020	Outcome	Trait, Choice, Action	UPPS-P, BIS/BAS, CCT, Flanker	Psychological, behavioral, cognitive outcomes	↑ prenatal alcohol exposure → ↑ impulsivity differences → ↑ problems, mediated by brain volume/area	Good
Marshall 2025	Outcome	Trait	UPPS-P, BIS/BAS	Prenatal tobacco/alcohol exposure, Cortical structure	↑ prenatal exposures → ↑ faster cortical thinning → ↑ impulsivity	Fair
Moore 2024	Predictor	Trait, Action	UPPS-P, BIS/BAS, Flanker, SST	Alcohol initiation, contextual factors	↑ impulsivity → ↑ alcohol use, but contextual factors explained more variance	Fair
Morys 2023	Mediator	Trait, Action	UPPS-P, Flanker	Body mass index change	↑ obesity PRS → ↓ cortical volume → ↑ impulsivity → ↓ body mass index change	Poor
Pang 2025	Predictor	Action	Flanker, SST	ADHD	↓ impulsivity (i.e., higher Flanker, lower SST) → ↓ ADHD symptoms	Good
Ortin-Peralta 2024	Mediator	Trait	UPPS-P	Suicide ideation & attempts	↑ parental suicide ideation/attempts → ↑ impulsivity → ↑ suicide ideation/attempts	Fair
Overholtzer 2025	Predictor	Choice	Delay discounting	Weight status, brain connectivity	↑ impulsivity → ↑ BMI and wait-to-height ratio	Good
Owens 2023	Correlate	Trait	UPPS-P	Neuroanatomical correlates	↑ impulsivity → brain structure associations	Fair
Scheve 2024	Predictor	Trait, Choice	UPPS-P, Delay discounting task	Suicidal ideation & attempts	↑ impulsivity → ↑ suicidal ideation/attempts	Good
Shao 2025	Predictor	Trait	BIS/BAS	Substance use initiation	↑ impulsivity → ↑ substance use risk	Fair
Smith 2023	Predictor, Moderator	Trait	UPPS-P, BIS/BAS	Binge eating disorder	↑ reward responsivity × negative urgency → binge eating disorder maintenance	Good
Song 2023	Outcome, Correlate	Trait	UPPS-P, BIS/BAS	Screen media activity patterns, functional connectivity	Screen use → ↑ impulsivity + altered functional connectivity	Good
Stinson 2024	Outcome	Trait, Action	UPPS-P, BIS/BAS, SST	Childhood adversity, inhibition BOLD signal	↑ adversity → altered neural activation → ↑ impulsivity	Good
Tan 2025	Outcome	Action	Flanker	Cognitive domains, physical activity	↑ sport participation → ↑ cognitive inhibition (↓ impulsivity)	Fair
Valdez 2023	Predictor	Trait	UPPS-P, BIS/BAS	Binge eating disorder	↑ impulsivity → ↑ body mass index	Good
Watts 2024	Predictor	Trait	UPPS-P, BIS/BAS	Alcohol sipping	↑ impulsivity → ↑ alcohol sipping	Good
Weigard 2023	Predictor	Trait, Choice, Action	UPPS-P, BIS/BAS, CCT, Flanker	ADHD symptoms	↑ impulsivity, cognition, screen time → ↑ ADHD symptom change	Fair
Weiss 2023	Predictor, Outcome	Trait	UPPS-P (Positive and negative urgency only)	Childhood trauma	Childhood trauma (T1) → ↑ Negative & Positive urgency (T2); ↑ Positive urgency (T1) → ↑ Childhood trauma (T2)	Fair
Wen 2023	Predictor	Trait	BIS/BAS	Self-injurious thoughts and behaviors	↑ impulsivity → ↑ transitions in self-injurious thoughts and behaviors	Fair
Wiglesworth 2024	Predictor	Choice, Action	Urgency (Negative & Positive), lack of premeditation; SST	Suicidal ideation	↑ impulsivity → ↑ suicidal ideation	Fair
Wojciechowski, (2024)	Mediator	Trait	UPPS-P	Deviant peer association, marijuana use	↑ Deviant peer association → (↑ Sensation seeking + ↓ lack of premeditation) → ↑ marijuana use	Good
Wojciechowski (2025a)	Predictor	Trait	UPPS-P	Marijuana use, parental monitoring	↑ sensation seeking + ↓ lack of premeditation → ↑ marijuana use risk; parental monitoring attenuated association	Fair
Wojciechowski (2025b)	Outcome	Trait	UPPS-P	Deviant peer association, age	↑ deviant peers → ↓ impulse control (no association with seeking, no moderation by age)	Fair
Wojciechowski (2025c)	Outcome	Trait	UPPS-P	Traumatic stress, deviant peer association	↑ traumatic stress → ↑ deviant peer association → ↓ impulse control, but no association with sensation seeking	Good
Wojciechowski (2025d)	Predictor	Trait	UPPS-P	Vaping onset, age	↑ sensation seeking + ↓ lack of premeditation → ↑ earlier vaping onset; age did not moderate	Good
Xu et al., 2025	Correlate	Trait	UPPS-P, BIS/BAS	Screen use, psychopathology	↑ mobile phone, social media → ↑ impulsivity; ↑ video games → ↓ impulsivity (for males)	Good
Yang et al., 2023	Predictor, Outcome	Trait	UPPS-P	Sleep, brain connectivity	Bidirectional - sleep duration ↔ urgency → corticostriatal connectivity → sleep/impulsivity	Good
Yin et al., 2025	Predictor, Outcome	Trait	UPPS-P	Family conflict, emotion regulation	↑ impulsivity ↔ ↑ family conflict, association attenuated at ↑ emotion regulation	Poor
Yin et al., 2025	Correlate	Action	Flanker Task	Executive functioning	Flanker impulsivity related to card and list tasks at baseline and less so 4 years later	Poor
Zhang et al., 2023	Outcome	Trait	UPPS-P	Sleep problems, duration	↑ Stress → ↑ sleep problems → ↑ impulsivity	Good
Zhang et al., 2025	Correlate, Outcome	Trait	UPPS-P	Sleep, rest-activity rhythms, school involvement	↑ Sleep timing delays → ↑ impulsivity	Fair
Zhang et al., 2025	Predictor	Trait	UPPS-P	Screen use	↑ impulsivity → ↑ mature movie watching	Fair

**Note**: ADHD = Attention-Deficit/Hyperactivity Disorder; BIS/BAS = Behavioral Inhibition System / Behavioral Activation System scales; studies using the BIS or BAS component alone were included in the BIS/BAS measure count; BMI = Body Mass Index; BOLD = Blood-oxygen-level–dependent (fMRI signal); CCT = Columbia Card Task; MDD = Major Depressive Disorder; PRS = Polygenic Risk Score; SST = Stop-Signal Task; T1 / T2 = Time 1 / Time 2; UPPS-P = Urgency, Premeditation, Perseverance, Sensation Seeking, Positive Urgency Impulsive Behavior Scale. ↑ = positive association; ↓ = negative association; ↔ = bidirectional relations.

### 4.2 Operationalization of impulsivity

Across the 51 included studies, impulsivity was most commonly operationalized using self-report trait measures. Forty-four studies (86.3%) included at least one self-report trait measure, including 42 (82.4%) studies using the UPPS-P or an urgency-specific measure and 23 (45.1%) using BIS/BAS scales. Among the 42 UPPS-P studies, facet coverage was relatively balanced across lack of premeditation or planning, urgency, sensation seeking, whereas lack of perseverance was examined somewhat less often (Table 3). Task-based impulsive choice and action measures appeared in 20 studies, including delay discounting, stop-signal, cash choice, and Flanker paradigms, and most often included as secondary predictors or examined alongside trait measures. Neuroimaging paradigms assessing impulsive action were used in a smaller subset of studies, though interpretation of task-based neural indices was often complicated by known psychometric limitations of these paradigms in large developmental samples (Bissett et al., 2021).

Critically, although all studies used two more ABCD data points and were longitudinal in nature, most operationalized impulsivity as a time-invariant construct, using measures from a single assessment rather than modeling trajectories or within-person change in impulsivity over time. Only two studies (Christopher-Hayes 2024; Song 2023) used growth curve, latent score change, or similar approaches to model developmental trajectories of impulsivity itself. However, with the most recent release (Release 7.0) of ABCD data and the ability to assess impulsivity development across a longer timespan (e.g., age 9–10 at baseline through year 7 when participants are 16–17 years old, and beyond), there will be opportunities for more evaluations of the repeated measures of trait impulsivity. Table 2, Table 3 summarizes study findings across self-report and task-based assessment in various health domains.

**Table 2:** Impulsivity as a predictor, outcome, and correlate across health domains in ABCD publications.

**Panel A – Studies using self-report impulsivity measures – UPPS-P; BIS/BAS**								
**Impulsivity Role**	**Substance Use**	**Mental Health / Social Risk Environment**		**Cognition / EF / Brain**	**Diet / Physical Health**	**Sleep / Screen use**		**Suicide Risk**
**Predictor**	Adams 2024 (T, ↑), Correa 2023 (T,↑), Moore 2024 (T, ↑), Kwon 2024 (T, ↑), Shao (T,↑),Watts 2024 (T,↑), Wojciechowski (2025a) (T,↑), Wojciechowski (2025d) (T, ↑),	Amir 2025 (T,↑), Chen 2025 (T, ↑), Correa 2023 (T,↑), Keyin 2025 (T, ↑), Smith 2023 (T, ↑), Weigard 2023 (T,↑), Weiss 2023 (T, ↑), Q. Q. Yin 2025 (T,↑),		Adise 2023a (T, ↑), Adise 2023b (T, ↑), Adise 2024 (T, ↑), Fan 2023 (T, ↑), Weigard 2023 (T,↑), Yang 2023 (T, ↑)	Adise 2023b (T, ↑), Adise 2024 (T, ↑), Smith 2023 (T, ↑), Valdez 2023 (T, ↑)	Grund 2025 (T,↑), Yang 2023 (T, ↑), L. Zhang 2025 (T, ↑)		Scheve 2024 (T,↑), Wen 2023 (T, ↑),
**Outcome**	Ferariu 2024 (T, ↑),	Gebru 2023 (T, ↑), Lees 2020 (T, ↑), Stinson 2024 (T, ↑), Weiss 2023 (T, ↑), Wojciechowski (2025b) (T, ↑), Wojciechowski (2025c) (T, ↑), Q. Yin 2025 (T, ↑)		Agarwal 2025 (T, ↑), Gilman 2024b (T, ↓), Lees 2020 (T, ↑), Marshall 2025 (T,↑), Song 2023 (T, ↑), Stinson 2024 (T, ↑), Yang 2023 (T ↑)	Adise 2023a (T, ↑)	Song 2023 (T, ↑), Yang 2023 (T ↑), Zhang 2023 (T, ↑)		
**Mediator/Moderator**	Wojciechowski, (2024) (T, ↑)	Amir 2025 (T,↑), Lahey 2022 (T, ↑)		Morys 2023 (T, ↓)	Morys 2023 (T,↑), Smith 2023 (T, ↑),	–		Ortin-Peralta 2024 (T, ↑)
**Correlate**	Adams 2024 (T, ↑), Gilman 2024a (T, ↓)	Fan 2023 (T, ↔), Gilman 2024b (T, ↑), Song 2023 (T, ↑)		Owens 2023 (T, ↑),		Xu 2025 (T, ↑), R. Zhang 2025 (T, ↑)		
**Panel B – Studies using task-based impulsivity measures – SST; Cash choice, DDT, Flanker**								
**Impulsivity Role**	**Substance Use**	**Psychopathology / Mental Health / Social Risk Environment**	**Cognition / EF / Brain**		**Diet / Physical Health**	**Sleep / Screen / Activity**	**Suicide Risk**	
**Predictor**	Adams 2024 (C, A, ↔), Ferariu 2024 (A, ↑), Kwon 2024 (A, ↑), Moore 2024 (A, ↑)	Gelino (C, ↑), Pang 2025 (A, ↑), Weigard 2023 (C, A,↑)	Weigard 2023 (C, A,↑), Wiglesworth 2024 (C,A,↑)		Adise 2023b (A, ↑) Gelino (C, ↑), Overholtz (C, ↑),	Gelino (C, ↑),	Wiglesworth 2024 (C,A,↑), Scheve 2024 (C, ↔)	
**Outcome**	–	Lees 2020 (C,A ↑)	Gilman 2024b (C, ↓), Lees 2020 (C,A, ↑), Tan 2025 (A, ↓)		Christopher-Hayes 2024 (A, ↓), Tan 2025 (A, ↓)	–		
**Mediator/Moderator**	–	Lahey 2022 (A, ↑)	Morys 2023 (A, ↓)		Morys 2023 (A, ↑)	–		
**Correlate**	–	–	R. Yin 2025 (A, ↑)		–	–		

**Note**: T = trait/self-report impulsivity (e.g., UPPS-P, BIS/BAS), A = Action (SST, Flanker), C = Choice (Cash, DDT); ↑ = positive association; ↓ = negative association; ↔ = null or mixed findings. Studies may appear in multiple domains when impulsivity was examined in relation to more than one outcome. Impulsivity roles reflect the analytic function of impulsivity in each study (predictor, outcome, mediator, moderator, or covariate), not assumed causal direction.

**Table 3:** Frequency of impulsivity measures and constructs across included studies.

**Measurement domain**	**Measure or construct**	**Studies, k**	**% of 51**	**% of 42 UPPS-P studies**
**Self-report trait**	Any self-report trait measure	44	86.3	-
	UPPS-P or urgency-specific measure	42	82.4	100.0
**UPPS-P facets/constructs**	Any urgency-related construct^1^	28	54.9	66.7
	Negative urgency	27	52.9	64.3
	Positive urgency	24	47.1	57.1
	Sensation seeking	27	52.9	64.3
	Lack of premeditation or planning	28	54.9	66.7
	Lack of perseverance	22	43.1	52.4
	UPPS-P composite only^2^	7	13.7	16.7
**Inhibition/reward sensitivity**	BIS and/or BAS scales	23	45.1	-
**Task-based**	Any task-based measure	20	39.2	-
**Impulsive action**	Any impulsive-action measure	16	31.4	-
	Flanker task	11	21.6	-
	Stop-signal task	7	13.7	-
	Little Man task	2	3.9	-
**Impulsive choice**	Any impulsive-choice measure	8	15.7	-
	Delay discounting task	4	7.8	-
	Cash Choice Task	3	5.9	-

**Note:** Categories are not mutually exclusive. ¹Includes studies examining negative urgency, positive urgency, or a combined urgency score. ²Composite-only studies analyzed an overall UPPS-P or trait-impulsivity score without reporting facet-specific associations. Percentages in the final column use the 42 studies that included the UPPS-P or an urgency-specific measure as the denominator. Constructs were counted at the study level when included in an analysis, rather than merely described as part of a measure. BIS/BAS = Behavioral Inhibition System/Behavioral Activation System.

### 4.3 Impulsivity as a predictor

Impulsivity was most commonly modeled as a predictor of later outcomes across multiple health domains.

#### 4.3.1 Substance use

Impulsivity was most frequently examined as a predictor of substance use initiation, escalation, and related outcomes. Urgency, sensation seeking, and lack of premeditation (impulse control) each showed prospective associations with substance use related outcomes, including alcohol sipping, earlier initiation or experimentation, and escalation (Adams et al., 2024, Grund and Luciana, 2025, Kwon et al., 2024, Moore et al., 2024, Shao et al., 2025, Watts et al., 2024). Beyond alcohol, impulsivity also predicted cannabis use (Wojciechowski, 2025a), vaping onset (Wojciechowski, 2025d), and cannabis harm perceptions (Gilman et al., 2024a). Studies using task-based measures found that dual-systems models incorporating both heightened reward sensitivity and reduced inhibitory control conferred increased substance use risk (Kwon et al., 2024, Moore et al., 2024, Weigard et al., 2023), though effect sizes were generally smaller and less consistent than those for self-report trait measures (Lees et al., 2020, Stinson et al., 2024).

#### 4.3.2 Psychopathology and mental health

Impulsivity was also modeled as a predictor in studies of internalizing and externalizing psychopathology and related mental health outcomes (Fan et al., 2023). Higher impulsivity predicted subthreshold depression severity (Chen et al., 2025), anxiety symptoms (Correa et al., 2023, Keyin et al., 2025), ADHD symptoms (Pang et al., 2025, Weigard et al., 2023), binge eating disorder (Smith et al., 2023), self-injurious thoughts and behaviors (Wen et al., 2023) and suicidal ideation (Scheve et al., 2024, Wiglesworth et al., 2024). Urgency-related traits were prospectively associated with several psychopathology related outcomes, including binge eating disorders, self-injurious thoughts and behaviors, and suicidal ideation or attempts. Behavioral inhibition and other impulsivity traits were also associated with anxiety and related psychopathological outcomes (Correa et al., 2023, Keyin et al., 2025). Correa et al. (2023) also examined whether the longitudinal association between behavioral inhibition and anxiety differed by Hispanic/Latinx ethnicity. Behavioral inhibition predicted greater anxiety symptoms at the one- and two-year follow-ups, but these associations did not differ significantly by ethnicity, suggesting behavioral inhibition as a prospective correlate of anxiety with no evidence for ethnicity moderation.

#### 4.3.3 Cognition, brain, and neuroimaging

A smaller subset of studies examined impulsivity as a predictor of neurocognitive or brain-related outcomes. Higher impulsivity was associated with reduced cortical thickness and surface area, accelerated cortical thinning, and alterations in fronto-temporal regions implicated in reward processing (Adise et al., 2023, Gilman et al., 2024b). Impulsivity also predicted functional connectivity and task-related neural activation within corticostriatal and frontoparietal networks (Yang et al., 2023). Neuroimaging associations were generally small in magnitude and distributed across regions rather than localized, consistent with contemporary neurodevelopmental accounts emphasizing broad maturational processes over discrete neural signatures. Several studies examined contextual influences on neural or neurocognitive outcomes. For example, greater childhood adversity was associated with reduced activation in regions implicated in inhibitory control (Adise et al., 2023b); however, family conflict did not significantly moderate these associations. These findings identify contextual correlates of inhibitory control circuitry but provide limited evidence regarding moderators of brain-impulsivity associations.

#### 4.3.4 Physical health, diet, sleep, and screen use

Impulsivity predicted weight-gain and body mass index (BMI)-related outcomes (Valdez et al., 2023) and dietary intake patterns (Adise et al., 2024), as well as sleep problems and screen/media use (Grund and Luciana, 2025, Yang et al., 2023; Zhang et al., 2025). Delay discounting predicted sleep and obesity-related outcomes, extending predictive utility of choice-based impulsivity measures beyond substance use contexts (Overholtzer et al., 2025).

### 4.4 Impulsivity as an Outcome

Several studies modeled impulsivity as a dependent variable, finding it was shaped by prenatal exposures to substances (Lees et al., 2020, Marshall et al., 2025); parental mental health and family conflict (Gebru et al., 2023; Yin et al., 2025), childhood adversity (Agarwal et al., 2025), traumatic stress (Wojciechowski 2025c), and deviant peer associations (Wojciechowski, 2025b). Bidirectional relations were also identified. Alcohol sipping at year 2 predicted urgency at year 4, and urgency predicted subsequent alcohol use (Ferariu et al., 2024), suggesting reciprocal developmental pathways between impulsivity and substance use behavior. Sports participation was associated with enhanced cognitive inhibition performance (Tan et al., 2025), pointing toward potential protective pathways. Taken together, these findings highlight that impulsivity is not a fixed trait, rather a developmentally malleable process responsive to environmental context.

### 4.5 Impulsivity as a mediator, covariate, or correlate

A smaller subset of studies examined mechanistic pathways. Impulsivity mediated associations between ADHD polygenic risk scores and psychological problems (Lahey et al., 2022); family conflict and suicide risk (Ortin-Peralta et al., 2024), peer exposure and cannabis use (Wojciechowski, 2024), brain structure/function and BMI changes (Morys et al., 2023), among others (Amir et al., 2026). Other studies included impulsivity as a covariate or correlate (e.g., Song et al., 2023), to control for reward responsiveness or baseline risk (Gilman et al., 2024a, Weiss et al., 2023) or assess measurement reliability (Owens et al., 2023).

### 4.6 Longitudinal modeling of impulsivity over time

Explicit statistical modeling intended to capture the progression of impulsivity across adolescence was rare across the reviewed literature. Only two studies used growth curve, latent change score, or related approaches to characterize developmental trajectories of impulsivity as the primary analytic focus (Christopher-Hayes 2024; Song 2023). These studies highlight that trajectory modeling of impulsivity facets is feasible within ABCD, although no consistent pattern of developmental change was identified. Song and colleagues’ (2023) investigation revealed that impulsive personality traits changed across adolescence, and developmental trajectories varied as a function of the screen media activity patterns, with high frequency users demonstrating higher impulsivity. On the other hand, Christopher-Hayes et al. (2024) examined inhibition and attention in youth with and without asthma and found improvement in inhibition and attention across development, although the rate of change was comparable across groups. These findings add to our understanding of the progression and development of impulsivity, especially in response to environmental or behavioral factors, although more explicit trajectory modeling across longer periods is needed to clarify developmental patterns.

## 5 Discussion

This review synthesizes findings from 51 longitudinal studies using data from the ABCD study to examine impulsivity across behavioral, psychological, neurodevelopmental, and other health domains during preadolescence and early adolescence. Taken together, three integrative conclusions emerge. First, ABCD studies extend prior evidence by demonstrating recurring prospective associations between multiple self-reported impulsivity facets and health risk across a large, demographically diverse multisite cohort. Second, task-based findings were generally less consistent than self-report findings, whereas the small number of eligible neuroimaging studies precluded reliable cross-modality comparisons. Third, this review captures the first decade of research from the ABCD Study, a period during which investigators have established foundational findings regarding impulsivity and its associations with health outcomes. This time frame also reflects relatively early stage of data availability with only a limited number of papers modeling impulsivity trajectories. As the ABCD cohort continues through adolescence and into early adulthood, and more data are released, modeling developmental trajectories represents a major opportunity for future research.

### 5.1 What ABCD adds and where it challenges prior work on impulsivity

Across the reviewed literature, self-reported impulsivity facets showed recurring prospective associations with health risk. Urgency-related traits were associated with substance use, psychopathology, and related outcomes, while sensation seeking and lack of premeditation (or impulse control) were also implicated across several behavioral and health domains. This pattern is consistent with prior research in clinical and community samples, including meta-analyses identifying urgency associated with psychopathology and substance-related problems (Berg et al., 2015, Coskunpinar et al., 2013, Hershberger et al., 2017). In the present review, lack of premeditation or planning, urgency, and sensation were examined with relatively similar frequency. Because the included studies differed in outcomes, follow-up periods, model specifications, and use of overlapping ABCD samples, the evidence does not permit a definitive ranking of facets, rather supports multiple dimensions of impulsivity as early-emerging prospective correlates of health risk during pre- and early adolescence. Prior research suggests that sensation seeking may be more closely related to experimentation or frequency of substance use, whereas urgency may be more strongly associated with problematic consequences, although these patterns vary across behaviors and developmental periods (Coskunpinar et al., 2013, Stautz and Cooper, 2013). In the future, longitudinal ABCD analyses could further test this potential specificity directly by comparing facets and clarify whether facet-specific differences precede, follow, or develop reciprocally with emerging risk behavior. Future multimethod studies could also determine whether self-reported facets, BIS/BAS, behavioral tasks, and neurobiological measures offer complementary information.

An area where the ABCD literature departs from prior work is in the signal from task-based measures. Delay discounting and stop-signal paradigms have shown strong associations with substance use and externalizing outcomes in smaller, often clinically enriched samples (MacKillop et al., 2016, Reynolds and Schiffbauer, 2004). In ABCD, these associations are present but smaller and less consistent. This attenuation in effects may reflect the population-level sampling strategy or the early developmental window, during which inhibitory control and delay of gratification are still maturing and behavioral variance in these measures may not yet translate into stable individual differences with strong predictive utility. This pattern aligns with longstanding psychometric critiques of task-based measures regarding test-retest reliability and convergent validity in large developmental samples (Bissett et al., 2021, Hedge et al., 2018, Strickland and Johnson, 2021). It could be the case that task-based measures in ABCD may be capturing meaningfully distinct facets of impulsivity from trait self-report measures, which would have implications for how these measures are used in developmental models of impulsivity and related constructs going forward.

### 5.2 Measurement heterogeneity and conceptual considerations

The dominance of self-report measures across the reviewed literature, particularly use of UPPS-P and BIS/BAS facets, reflects both their psychometric strengths and practical advantages in large-scale developmental research, particularly with younger participants. These measures are well-validated, sensitive to individual differences, and consistently linked to outcomes. Critically, these measures do not depend on scoring conventions that can meaningfully alter what is being indexed. Task-based impulsivity measures, by contrast, involve analytic decisions that vary across studies (e.g., how to determine non-systematic responding on delay discounting tasks) and may affect the reliability and validity of derived indices, and resulting interpretations. For the delay discounting task in the ABCD literature specifically, a study included in this review (Gelino et al., 2025) found that responses typically classified as non-systematic, and thus often excluded from analysis, may themselves have predictive signal rather than representing noise. This challenges the standard practice of exclusion and raises the possibility that current scoring conventions may be systematically missing meaningful variance. Analogous issues are present for other tasks, including the Stop-Signal and Flanker paradigms, where the methods in preparing and scoring data affects the magnitude and direction of associations with outcomes. Taken together, these scoring considerations may partly explain why task-based findings in ABCD are less consistent across studies than self-report findings.

Given the importance of task measures in capturing impulsive action and choice that self-report cannot assess, future research can continue to assess and refine scoring methods. Future meta-analyses may also require standardization of scoring conventions across ABCD-based studies to allow meaningful cross-study synthesis. Future ABCD-based research can also integrate both self-report and task-based modalities within the same analytic models, and test whether they predict outcomes independently or interactively, which would substantially advance understanding of which impulsivity (and impulsivity-adjacent) processes are most developmentally impactful.

### 5.3 The developmental gap

The use of two or more waves of ABCD data was an inclusion criterion for this review. Despite the longitudinal design being ABCD’s defining methodological asset, only a small number of studies to date included explicitly modeled change in impulsivity over time using growth curve, latent change, or similar approaches. The vast majority of studies used earlier impulsivity scores to predict later health outcomes; a prospective design that is valuable but does not address how impulsivity itself develops. As more ABCD data are released, as the youth are getting older, trajectory modeling of specific impulsivity facets across the full preadolescent-to-adolescent window will be feasible, and within-person change analyses could begin to disentangle cause from consequence in impulsivity-risk pathways. Studies that modeled impulsivity as an outcome found it shaped by stress, family conflict, peer associations, and prenatal exposure. This work provides initial evidence of developmental malleability that trajectory-focused work could formalize.

### 5.4 Effect sizes and implications for interpretation

Statistically significant findings were common across the reviewed studies, although reported associations were generally modest in magnitude. Several studies reported correlations and standardized regression coefficients with absolute values below .15, particularly for task-based and neuroimaging predictors, however effect estimates are interpreted in relation to their analytic model, uncertainty, outcome and cumulative consistency rather than statistical significance or a single universal threshold. This is perhaps unsurprising given ABCD’s large sample size, where modest effects are often expected (Owens et al., 2021). Small effect sizes are not uncommon among developmental research with large samples and suggest that results should be interpreted as identifying probabilistic contributors to risk rather than strong individual-level predictors. At the population scale, modest associations involving widely distributed risk factors may nevertheless have public health relevance. However, repeated findings produced by different analytic teams using the same ABCD cohort should be understood as evidence of reproducibility across analytic specifications rather than independent replication across samples.

### 5.5 Implications for future research

Findings from the current review support several strategic future directions. Trajectory modeling of impulsivity facets, including urgency, sensation seeking, and lack of premeditation or planning across ABCD waves is needed to establish normative and maladaptive developmental patterns and identify periods of heightened malleability. Greater harmonization of scoring and reporting conventions across ABCD studies, particularly of task-based paradigms, would enable meaningful cross-study comparison, which is currently precluded by analytic heterogeneity. ABCD’s breadth offers unprecedented promise to integrate behavioral, neural, and contextual data, rather than in separate literatures, which will advance our understanding of whether these modalities index shared or distinct developmental processes. As participants age into peak risk periods for substance use and psychopathology, the predictive validity of early impulsivity measures with later-wave outcomes should be tested, including whether small early effects strengthen, attenuate, or are moderated/mediated by intervening experiences. Contextual factors, including adversity, family environment, and peer influences, were associated with impulsivity or related outcomes across several studies, although evidence of statistical moderation was limited. Future studies should test theoretically specified interactions, report simple slopes or conditional effects, and examine whether contextual factors amplify or attenuate impulsivity-related risk across development.

From a public health perspective, these results support prevention strategies that target shared self-regulatory processes rather than disorder-specific outcomes. Because impulsivity is shown to both predict later risk and be shaped by environmental exposures, interventions that strengthen emotional regulation, reduce chronic stress, and improve caregiver and school supports during preadolescence may mitigate downstream substance use and mental health problems. Results also suggest prevention strategies that address modifiable environmental processes, rather than treating impulsivity as a fixed individual characteristic, may be more efficacious.

### 5.6 Strengths and limitations

This review has several strengths. The search was anchored to the complete ABCD publication archive and peer-reviewed by a second librarian, minimizing the risk of missing eligible studies. The focus on longitudinal studies only, requiring two or more ABCD waves, ensures that included findings reflect temporal relationships rather than cross-sectional associations. The requirement that studies analyze at least two ABCD waves to be eligible for this review may also have affected the representation of neuroimaging research. Tabulated imaging-derived variables (i.e., preprocessed neuroimage variables) became available longitudinally in later data releases than many questionnaire and behavioral variables. For instance, the ABCD 5.0 release in July 2023 contained the first full-cohort two-year neuroimaging follow-up data. Moreover, investigators conducting specific functional connectivity and/or structural analyses may need to process large raw imaging datasets, creating computational demands that can delay or constrain multi-wave analyses. Consequently, the small number of eligible neuroimaging studies may partly reflect data-release timing and analytic burden rather than limited scientific interest or predictive utility. Study conclusions therefore apply specifically to the longitudinal evidence meeting review criteria and should not be generalized to the broader ABCD neuroimaging literature. The included studies span diverse health domains, providing a comprehensive account of impulsivity (and impulsivity-adjacent) facets have been examined across in the first 10 years of the ABCD study, and informs future directions for the next decade.

Limitations include risk of publication bias toward statistically significant findings. Direct quantitative comparisons across studies were precluded due to wide heterogeneity in operationalizations of impulsivity, analytic approaches, and outcome measurement. The sample’s current developmental stage, being largely in the preadolescent phase, constrains outcome variability for certain outcomes (e.g., substance use), and some associations may strengthen as participants age. Heavy reliance on self-report measures, limited trajectory modeling of change over time, and uneven coverage across outcome domains all reflect the current state of the literature rather than decisions of this review, but they remain important interpretive constraints and important areas for future research.

## 6 Conclusion

This review demonstrates that ABCD-based research has identified multiple self-reported impulsivity facets as early emerging prospective correlates of health risk across a large developmental cohort of preadolescents. It has also revealed that the longitudinal potential of the dataset has not yet been fully realized, with most studies treating impulsivity as stable predictor rather than a developmental process. As additional ABCD data waves become available, the field is well-positioned to move from establishing associations to modeling mechanisms, trajectories, and points of intervention.

## CRediT authorship contribution statement

**Nioud Mulugeta Gebru:** Writing – review & editing, Writing – original draft, Visualization, Validation, Supervision, Resources, Project administration, Methodology, Investigation, Formal analysis, Data curation, Conceptualization. **Priscila Dib Gonçalves:** Writing – review & editing, Writing – original draft, Methodology, Investigation, Formal analysis. **Faith Adams:** Writing – review & editing, Writing – original draft, Methodology, Investigation, Data curation. **Nicita Anand:** Writing – review & editing, Data curation. **Laura N. Haygood:** Writing – review & editing, Writing – original draft, Methodology, Data curation. **Micah E. Johnson:** Writing – review & editing, Writing – original draft. **Alexandra O. Cohen:** Writing – review & editing, Writing – original draft, Methodology, Investigation, Data curation.

## Funding

This work was supported by the 10.13039/100000002National Institutes of Health: R00AA031443 (Gebru), K01DA057389 (Goncalves), K01DA053438 (Cohen), 1K01DA052679 (Johnson), R25DA059073 (Johnson, MPI) as well as NARSAD/Brain Behavior Research Foundation (Goncalves).

## Declaration of Competing Interest

The authors declare the following financial interests/personal relationships which may be considered as potential competing interests:Gebru reports financial support was provided by National Institutes of Health. Dib Goncalves reports financial support was provided by National Institutes of Health. Dib Goncalves reports financial support was provided by Brain and Behavior Research Foundation. Cohen reports financial support was provided by National Institutes of Health. Johnson reports financial support was provided by National Institutes of Health. If there are other authors, they declare that they have no known competing financial interests or personal relationships that could have appeared to influence the work reported in this paper.

## Data Availability

No data was used for the research described in the article.

## Footnotes

Appendix A Supplementary data associated with this article can be found in the online version at doi:10.1016/j.dcn.2026.101832.
