# Supplementary material for: Impulsivity during adolescent development: Insights from a decade of ABCD study longitudinal analyses
Source: Dev Cogn Neurosci. 2026 Oct 6;82:101832. doi: 10.1016/j.dcn.2026.101832 (PMC13694974; doi:10.1016/j.dcn.2026.101832)
Supplement: Supplementary file 1 [file mmc1.docx]

# ABCD Study and Impulsivity Search Report

Final comprehensive search. Inclusive of publications through July 2025 with the addition of tasks-related search terms.

| Database | MEDLINE | |
| --- | --- | --- |
| Platform | Ovid | |
| Date of last search | May 20, 2026 | |
| Number of results | 190 | |
| Notes | Ovid MEDLINE(R) ALL <1946 to May 19, 2026> | |
|  |  |  |
| 1 | ("40707713" or "40706587" or "40699480" or "40694209" or "40681863" or "40675965" or "40680997" or "40652980" or "40644691" or "40617802" or "40617820" or "40627885" or "40623653" or "40645110" or "40601692" or "40603722" or "40595447" or "40605715" or "40628004" or "40699580" or "40583545" or "40583442" or "40560555" or "40578581" or "40555602" or "40614528" or "40605961" or "40651096" or "40549408" or "40548973" or "40549406" or "40578273" or "40542091" or "40536230" or "40531495" or "40531519" or "40526716" or "40584278" or "40495526" or "40514009" or "40537387" or "40474740" or "40460372" or "40456721" or "40457117" or "40459483" or "40466988" or "40476050" or "40494109" or "40540271" or "40356347" or "40444692" or "40442425" or "40440273" or "40483797" or "40432344" or "40479751" or "40487008" or "40399342" or "40409705" or "40625792" or "40382469" or "40382724" or "40379614" or "40496708" or "40383189" or "40368909" or "40413902" or "40561462" or "40367732" or "40350165" or "40346141" or "40359598" or "40366339" or "40332609" or "40329546" or "40328752" or "40323157" or "40323914" or "40334862" or "40319141" or "40334044" or "40232785" or "40265644" or "40384091" or "40397441" or "40364472" or "40377939" or "40319611" or "40302646" or "40530340" or "40365462" or "40293811" or "40303901" or "40281225" or "40286517" or "40280216" or "40273521" or "40347767" or "40261403" or "40274111" or "40263586" or "40288104" or "40268244" or "40519635" or "40244849" or "40511327" or "40567457" or "40228565" or "40222467" or "40222403" or "40210909" or "40205758" or "40193606" or "40194858" or "40189068" or "40186956" or "40186073" or "40181189" or "40172268" or "40242473" or "40147852" or "40211548" or "40227687" or "40238096" or "40277222" or "40156904" or "40152459" or "40156962" or "40140344" or "40136996" or "40130379" or "40125918" or "40126000" or "40128186" or "40303380" or "40114533" or "40118942" or "40114020" or "40114021" or "40139048" or "40102735" or "40120644" or "40107499" or "40087503" or "40087898" or "40090057" or "40096952" or "40090385" or "40117703" or "40071551" or "40144916" or "40065572" or "40063275" or "40149652" or "40080996" or "40166767" or "40044931" or "40038058" or "40034088" or "40028695" or "40108831" or "40396529" or "39915975" or "40086410" or "40014542" or "40023259" or "40000536" or "39992757" or "39984505" or "39988295" or "39979383" or "40226806" or "39976710" or "39966895" or "39966400" or "40041111" or "39957496" or "39983553" or "39945682" or "39929394" or "39999579" or "39923932" or "40003454" or "39921132" or "39905395" or "39818428" or "39893243" or "39914190" or "39876084" or "39892220" or "39870773" or "39869720" or "39864234" or "39864502" or "39843235" or "39843416" or "39834255" or "39831924" or "39836167" or "39836146" or "39842629" or "39827443" or "39821510" or "39842631" or "39818916" or "39818657" or "39848135" or "39800219" or "39786680" or "39798665" or "39760735" or "39760238" or "39749564" or "39754988" or "39743556" or "39746839" or "39788921" or "39844367" or "39818928" or "39865519" or "39736052" or "39724179" or "39701594" or "39740341" or "39706190" or "39724816" or "39729899" or "39701413" or "39689948" or "39708510" or "39673771" or "39668158" or "39735971" or "39722687" or "39657055" or "39656219" or "39662779" or "39630417" or "39656842" or "39638016" or "39638054" or "39625475" or "39622794" or "39699892" or "39656455" or "39786411" or "39786408" or "39587984" or "39616272" or "39614397" or "39605296" or "39604734" or "39613384" or "39587853" or "39587556" or "39600305" or "39601720" or "39592017" or "39578521" or "39570768" or "39629197" or "39552379" or "39566883" or "39566882" or "39546599" or "39612721" or "39545896" or "39541797" or "39526341" or "39520143" or "39537024" or "39504015" or "39505862" or "39520899" or "39748875" or "39816599" or "39496559" or "39514229" or "39527060" or "39501896" or "39526419" or "39531710" or "39488236" or "39522301" or "39475730" or "39541739" or "39490580" or "39441608" or "39440454" or "39447970" or "39488011" or "39434468" or "39433889" or "39488499" or "39415428" or "39404167" or "39563872" or "39397679" or "39484302" or "39390801" or "39394256" or "39389909" or "39385600" or "39763511" or "39384894" or "39381955" or "39384554" or "39389112" or "39370520" or "39507937" or "39468741" or "39343719" or "39398321" or "39324400" or "39324563" or "39324385" or "39341186" or "39368282" or "39376969" or "39306006" or "39385747" or "39323982" or "39288146" or "39289875" or "39283646" or "39279127" or "39276595" or "39303554" or "39253855" or "39246252" or "39251706" or "39315000" or "39244671" or "39240900" or "39245397" or "39242605" or "39241464" or "39243820" or "39234169" or "39231917" or "39232159" or "39226358" or "39243851" or "39283634" or "40519960" or "39223252" or "40519962" or "39215207" or "39279846" or "39196567" or "39197577" or "39179884" or "39181217" or "39171674" or "39243745" or "39196964" or "39161279" or "39232266" or "39154134" or "39150663" or "39154835" or "39140927" or "39140868" or "39136075" or "39140930" or "39153718" or "39134208" or "39153717" or "39262669" or "39127423" or "39113318" or "39170667" or "39106155" or "39097657" or "39816605" or "39025633" or "39090375" or "39085406" or "39085394" or "39072760" or "39084501" or "39060518" or "39070965" or "39089096" or "39128397" or "39184526" or "39031509" or "39099631" or "39018056" or "39029692" or "39020167" or "39009591" or "39061446" or "39009188" or "39009136" or "38996031" or "38996016" or "38997510" or "39004299" or "39001748" or "39046391" or "38980723" or "39001757" or "38982203" or "39697392" or "38956310" or "38932715" or "38952810" or "38967921" or "39073774" or "38915228" or "38988766" or "38902353" or "38898062" or "38890123" or "38889233" or "38924835" or "39175501" or "38879625" or "38905775" or "38878049" or "38874007" or "38869879" or "38909414" or "38851850" or "38852089" or "38844772" or "38834780" or "38852932" or "38849031" or "38840198" or "38850213" or "38880786" or "38870601" or "38829657" or "38865127" or "38865126" or "38821981" or "39021436" or "38814823" or "38806652" or "38807026" or "38809249" or "38803212" or "38823157" or "38838435" or "38779781" or "38780543" or "38778392" or "38771191" or "38762449" or "38827440" or "38759754" or "38748406" or "38740587" or "38781677" or "38749217" or "38717122" or "38713673" or "38775636" or "38710591" or "38719179" or "38718977" or "38706327" or "38717776" or "38684513" or "38870743" or "38677137" or "38663994" or "38662452" or "38721152" or "39464493" or "38676989" or "38632388" or "38618861" or "38626612" or "38609424" or "38615556" or "38602745" or "38605111" or "38626611" or "38589467" or "38593638" or "38603863" or "38588854" or "39242809" or "38572625" or "38575908" or "38623304" or "38566134" or "38583301" or "38479979" or "38520363" or "38530121" or "38553865" or "38590252" or "38669008" or "38532735" or "38532493" or "38532024" or "38531894" or "38519749" or "38522613" or "38522612" or "38508874" or "38503761" or "38518431" or "38503979" or "38497117" or "38508468" or "38491089" or "38483518" or "38550535" or "38483374" or "38478161" or "38480986" or "38476041" or "38467686" or "38458308" or "38461893" or "38441539" or "38433592" or "38477919" or "38060253" or "38425212" or "39242757" or "38418819" or "38423255" or "38413633" or "38417787" or "38409281" or "38827988" or "38388427" or "38440191" or "38439791" or "38362835" or "38366843" or "38359015" or "38358046" or "38426165" or "38401329" or "38367454" or "38447469" or "38340416" or "38336620" or "38340122" or "38354531" or "38148152" or "38321588" or "38323959" or "38486948" or "38314519" or "38349651" or "38407905" or "38307840" or "38308475" or "38305870" or "38311068" or "38339899" or "38339910" or "38846637" or "38292554" or "38286629" or "38307126" or "38273035" or "38266514" or "38272350" or "38277713" or "38237797" or "38227391" or "38286089" or "38335828" or "38190059" or "38444906" or "38192094" or "38185921" or "38219709" or "38182882" or "38184855" or "38195369" or "38172629" or "38165022" or "38030307" or "38160517" or "38151182" or "38154377" or "38248230" or "38312852" or "38110396" or "38124022" or "38103132" or "38102419" or "38102486" or "38093235" or "38190638" or "38081908" or "38187352" or "38066316" or "38144472" or "38060365" or "38070629" or "38055638" or "38052980" or "38052266" or "38044723" or "38091780" or "38032600" or "38032599" or "38032598" or "38042404" or "38036780" or "38015355" or "37992006" or "37995848" or "38026821" or "37975498" or "37973980" or "37977408" or "37935035" or "37936270" or "37944709" or "37930714" or "37939873" or "37916265" or "37916784" or "37916808" or "37930702" or "37955897" or "37902502" or "37898383" or "37898357" or "37877924" or "37942273" or "37872174" or "38027832" or "37866541" or "37973444" or "37976871" or "37928910" or "37891836" or "37855753" or "37840377" or "37843837" or "37866325" or "37839556" or "37833810" or "37828061" or "37891820" or "37815218" or "37816912" or "37815759" or "37804305" or "37803213" or "37867762" or "37792148" or "37827934" or "37788383" or "37791924" or "37787573" or "37578771" or "38021250" or "37756262" or "37750998" or "37769982" or "37769983" or "37748537" or "37730654" or "37766729" or "37731207" or "37717292" or "37702839" or "37704547" or "37522299" or "37671456" or "37673436" or "37665306" or "37690156" or "37711221" or "37616102" or "37666116" or "37592024" or "37592364" or "37675438" or "37580525" or "37582185" or "38263994" or "37565595" or "37598455" or "37548898" or "37550530" or "37542243" or "37531585" or "37527347" or "37536527" or "36869122" or "37358866" or "37583413" or "37517645" or "37500827" or "38584801" or "37564120" or "37466292" or "37499380" or "37460809" or "37437033" or "37438345" or "37492602" or "37420169" or "37473699" or "37441978" or "37410001" or "37422106" or "37440345" or "37432683" or "37399053" or "37389520" or "37393046" or "37393047" or "37376848" or "38054056" or "37365781" or "37355620" or "37353663" or "37400062" or "37349707" or "37427258" or "37343155" or "37339753" or "37312172" or "37307315" or "37277147" or "38774602" or "37295068" or "37333663" or "37272416" or "37273065" or "37277504" or "37264939" or "37270396" or "37017952" or "37074691" or "37212029" or "37368403" or "37307604" or "37248201" or "37237028" or "37236919" or "37227824" or "37313357" or "37202553" or "37210754" or "37200192" or "37191599" or "37201711" or "37183911" or "37182734" or "37178821" or "37164954" or "37165514" or "37196505" or "37233339" or "37251494" or "36573454" or "37155868" or "37146216" or "37141274" or "37130880" or "37207193" or "37095251" or "37200030" or "37200032" or "37119331" or "37119410" or "37121399" or "37106035" or "37126727" or "37129746" or "37095243" or "37119589" or "36255322" or "37093311" or "37095248" or "37098449" or "37088322" or "37076628" or "37071275" or "37062398" or "37053187" or "37046279" or "37039543" or "37036551" or "37037991" or "37024669" or "37032213" or "37031778" or "37023280" or "37017779" or "37013949" or "37012426" or "37115542" or "37084569" or "37003411" or "36972087" or "37023632" or "36951577" or "36965838" or "36950909" or "36946606" or "36946069" or "36963562" or "35989308" or "36948435" or "36944127" or "36940062" or "36940760" or "36940096" or "36939078" or "36928354" or "37753156" or "36934605" or "36947895" or "36912775" or "36918134" or "36918136" or "36906575" or "35697648" or "36933272" or "36969492" or "38405128" or "36893272" or "36890392" or "36882872" or "36879232" or "36853039" or "36872118" or "36890374" or "36912834" or "36930149" or "36995714" or "36965337" or "36840387" or "36870214" or "35104344" or "36841180" or "36908780" or "36805495" or "36875509" or "36801369" or "37881576" or "36821878" or "36795263" or "36765403" or "37881582" or "36757558" or "36773698" or "36746929" or "36791556" or "34216137" or "34471927" or "36318557" or "36722118" or "36722124" or "36757695" or "36795417" or "36809470" or "36915692" or "36720770" or "36716140" or "36705774" or "36706638" or "36833216" or "36689685" or "36662388" or "36662346" or "36653486" or "36652896" or "36633989" or "36630774" or "36609385" or "36621021" or "36596778" or "36812240" or "36610772" or "36577839" or "36581100" or "36672028" or "36562588" or "36580867" or "36603413" or "36542658" or "36534603" or "36567744" or "36523258" or "35238352" or "36528521" or "36589997" or "36517639" or "36517380" or "36482079" or "38818252" or "37431413" or "36473996" or "36745173" or "36469439" or "36094599" or "36456690" or "36440655" or "36442048" or "36443937" or "36495789" or "36450307" or "37881561" or "36501894" or "36552062" or "36424333" or "36408958" or "36436429" or "35894236" or "36385331" or "36384578" or "36777645" or "36379997" or "36405885" or "36380235" or "36687306" or "36400261" or "37780352" or "36305770" or "36357558" or "36355697" or "36350121" or "36329481" or "36364905" or "36445708" or "36094004" or "36368089" or "36305572" or "36389240" or "36301559" or "36273036" or "36268881" or "36262017" or "36253967" or "36274574" or "36243789" or "36229943" or "36226844" or "36797123" or "36270101" or "36279138" or "35913732" or "36044238" or "36177528" or "36291253" or "36169746" or "36225678" or "36329717" or "36609028" or "36151456" or "36291360" or "36561641" or "36114937" or "36116616" or "36115590" or "36186872" or "36100657" or "36100668" or "36103956" or "36357217" or "36090673" or "36070838" or "35104356" or "36084446" or "35171285" or "36043323" or "36028495" or "36046104" or "35997913" or "35998220" or "35987133" or "35946823" or "35944262" or "35963164" or "36003980" or "37519454" or "35934545" or "36002962" or "35917308" or "35954804" or "35947383" or "35980639" or "35914537" or "35903877" or "35899848" or "35881083" or "35867480" or "35852028" or "35858412" or "35907312" or "35884741" or "35853601" or "35840085" or "35843514" or "35884720" or "35037940" or "35845820" or "35790754" or "35803444" or "35867063" or "35895476" or "35768491" or "35764363" or "35748113" or "35554607" or "35728730" or "35752070" or "35697691" or "35716638" or "35654767" or "35475851" or "35648742" or "35759263" or "36325038" or "34968177" or "35648269" or "35609016" or "35608364" or "35596841" or "35636343" or "35594974" or "35579325" or "34664626" or "37519461" or "35552993" or "35545630" or "35545159" or "35531607" or "35624986" or "35525409" or "35505052" or "35452534" or "35509143" or "35471639" or "35483606" or "35468875" or "35436615" or "35427796" or "35427730" or "35393594" or "35388223" or "35383335" or "35135639" or "35138333" or "35378237" or "35380644" or "35396019" or "34382607" or "35361904" or "35431841" or "35431847" or "35341934" or "35307818" or "35220581" or "35301976" or "35314358" or "35296861" or "35294251" or "35294630" or "35344870" or "35181902" or "35238432" or "35360125" or "35249585" or "35308882" or "35489875" or "35445220" or "35239427" or "35147993" or "35232507" or "35248457" or "35229959" or "35245674" or "37124359" or "35212098" or "35217109" or "35217219" or "37124351" or "35220054" or "35248821" or "35172771" or "36387418" or "35141898" or "35223717" or "35132700" or "35136189" or "34379727" or "34467389" or "35143759" or "36187359" or "35152002" or "35113692" or "35123109" or "35186306" or "35188556" or "35101172" or "35023210" or "35144197" or "35123342" or "35090817" or "35075111" or "35072847" or "35067249" or "35064714" or "35078736" or "35199043" or "35061153" or "35075340" or "35048539" or "35040524" or "36712562" or "35034850" or "35145385" or "35033950" or "37323064" or "35026661" or "35052446" or "34724543" or "34432631" or "35004134" or "35216811" or "34954668" or "34952233" or "34920689" or "36090949" or "34923146" or "34914494" or "37449490" or "34920091" or "34916124" or "34893612" or "34915436" or "34845019" or "36238203" or "35036918" or "34860306" or "34896850" or "36339462" or "34882178" or "34263059" or "34323206" or "34307699" or "34899225" or "34823023" or "34849476" or "34802858" or "37310327" or "34782711" or "34795422" or "35027165" or "34753911" or "34750359" or "34891080" or "34781251" or "34734493" or "34790165" or "34267084" or "34742018" or "34765698" or "35047823" or "34690348" or "34695568" or "34677743" or "34661541" or "34710799" or "34700197" or "34648580" or "34650205" or "34607958" or "34692610" or "34605288" or "32522303" or "32654667" or "34599145" or "34608463" or "34555056" or "34552053" or "34560273" or "34661044" or "37310323" or "34555784" or "34594183" or "34496002" or "34496065" or "34506917" or "34506929" or "34486481" or "34481917" or "34481807" or "34452728" or "34468031" or "34530359" or "33987673" or "34425231" or "34419766" or "35677113" or "34392161" or "33938908" or "33999104" or "34288270" or "34392051" or "34318489" or "34332330" or "34335337" or "34271214" or "34247260" or "33825852" or "34225382" or "33774662" or "35419552" or "34224907" or "33881474" or "34246036" or "34184767" or "34180585" or "34155395" or "34145259" or "34147629" or "34140357" or "34099922" or "34092032" or "34086035" or "34307955" or "34043478" or "35402089" or "34033672" or "34082235" or "34071105" or "34008881" or "34070118" or "34070074" or "34070158" or "33990770" or "34040634" or "33971956" or "34025378" or "33964518" or "34025573" or "33749724" or "33909652" or "33900639" or "33981668" or "33895776" or "33984711" or "33851904" or "33850154" or "33846287" or "33836108" or "33821002" or "33821375" or "33369616" or "33789457" or "34095893" or "33626946" or "33862325" or "34604532" or "33771979" or "33771727" or "33809905" or "33740410" or "33737246" or "33799042" or "33706021" or "33661097" or "33806587" or "33646623" or "33646417" or "33638150" or "33630070" or "33713937" or "33679599" or "33676072" or "34621600" or "33657461" or "33145600" or "33705164" or "33523708" or "33165022" or "33529676" or "33556882" or "33479512" or "33472387" or "33410532" or "33462190" or "33536880" or "33467473" or "33441214" or "33434614" or "33404947" or "33403494" or "32737734" or "32965490" or "33359407" or "33325561" or "33360278" or "33176359" or "33317053" or "33297546" or "33294964" or "33238925" or "33518499" or "33233814" or "33282415" or "33229052" or "33495121" or "33181393" or "32591777" or "33141160" or "33141156" or "33135469" or "33192411" or "33127479" or "33152602" or "33096046" or "33163684" or "33069581" or "33046629" or "33103157" or "32985363" or "32972200" or "32976083" or "33229246" or "32926284" or "33281105" or "32897083" or "32886714" or "32511674" or "34036110" or "32867775" or "32865228" or "32861729" or "33109338" or "32841716" or "32795607" or "32764344" or "32832919" or "32731811" or "32814618" or "32718077" or "32731083" or "32682894" or "32672986" or "32674966" or "32659528" or "32660094" or "32603213" or "32601990" or "32605891" or "32607126" or "32593800" or "32592852" or "32575523" or "32656052" or "32541809" or "32803159" or "32522466" or "32503310" or "33215045" or "32463952" or "32451322" or "32455841" or "32443584" or "32333792" or "32409600" or "32399985" or "31258099" or "31646343" or "32439147" or "32414481" or "32354687" or "32171431" or "32105123" or "32102994" or "32046896" or "32031652" or "32015467" or "31816020" or "31983035" or "32144045" or "31932788" or "31932789" or "31931163" or "31841018" or "31062126" or "31778819" or "31727084" or "31818798" or "31634568" or "31624235" or "31554938" or "31614255" or "31305867" or "31384026" or "31427753" or "31413180" or "31415884" or "31288867" or "30916716" or "30865236" or "31110341" or "31034667" or "30950740" or "30949565" or "30846008" or "30905689" or "30850130" or "30476983" or "30578952" or "30595399" or "30208469" or "30339913" or "30268792" or "30268791" or "29874361" or "29773510" or "29680211" or "29703560" or "29706313" or "29655614" or "29636283" or "29627333" or "29606560" or "29567376" or "29460349" or "29460352" or "29525452" or "29559216" or "29496476" or "29311006" or "29150307" or "29113758" or "29051027" or "29107609" or "28716389" or "29038777").ui. | 1445 |
| 2 | exp impulsive behavior/ | 29816 |
| 3 | (impuls* or urgency or sensation seeking or perseverance or planning or premeditation or inhibition or disinhibition or UPPS).ti,ab,kf. | 1588210 |
| 4 | (No-Go Task or ("Stop-Signal" adj2 Time) or SSRT or Continuous Performance Task or CPT or 5-Choice Serial Reaction Time Task or 5-CSRTT or Delay Discounting Task or Marshmallow Test or Iowa Gambling Task or IGT or Balloon Analogue Risk Task or BART or "Stroop Color and Word Test" or Matching Familiar Figures Task or MFFT or "Immediate and Delayed Memory Task" or "IMT/DMT" or Cash Choice Task or Flanker Task or Monetary Incentive Delay Task or MID Task or MIDT or "Reward Responsiveness Task" or Probabilistic Reward Task or PRT or Risk-taking task or Columbia Card Task or "Effort expenditure for rewards task" or EEfRT or Digit Span Task or Cambridge Gambling Task or CGT or Cognitive Reflection Test or CRT or Cups Task or Information Sampling Task or N-back Task or Little Man Task or Dot-probe task).ti,ab,kf. | 65156 |
| 5 | or/2-4 | 1664063 |
| 6 | 1 and 5 | 190 |
